# Supplementary material for: Impact of Magnetite Nanowires on In Vitro Hippocampal Neural Networks
Source: Biomolecules. 2023 Apr 30;13(5):783. doi: 10.3390/biom13050783 (PMC10216447; doi:10.3390/biom13050783)
Supplement: Supplementary file 1 [file biomolecules-13-00783-s001.zip › biomolecules-2317845-supplementary.pdf]

biomolecules

MDPI

Supplementary Materials

Impact of Magnetite Nanowires on In Vitro Hippocampal Neural Networks

Belén Cortés-Llanos [1,2,3,*], Rossana Rauti [4,5], Ángel Ayuso-Sacido [6,7], Lucas Pérez [1,2] and Laura Ballerini [4,*]

[1] Departamento de Física de Materiales, Universidad Complutense de Madrid, 28040 Madrid, Spain
[2] Fundación IMDEA Nanociencia, C/Faraday 9, 28049 Madrid, Spain
[3] Department of Medicine, Duke University, Durham, NC 27705, USA
[4] International School for Advanced Studies (ISAS-SISSA), 34136 Trieste, Italy
[5] Deparment of Biomolecular Sciences, Università degli Studi di Urbino Carlo Bo, 61029 Urbino, Italy; rossana.rauti@uniurb.it
[6] Brain Tumor Laboratory, Fundación Vithas, Grupo Hospitales Vithas, 28043 Madrid, Spain; ayusoa@vithas.es
[7] Faculty of Experimental Science and Faculty of Medicine, University of Francisco de Vitoria, 28223 Madrid, Spain
* Correspondence: belencortesllanos@gmail.com (B.C.-L.); ballerin@sissa.it (L.B.)

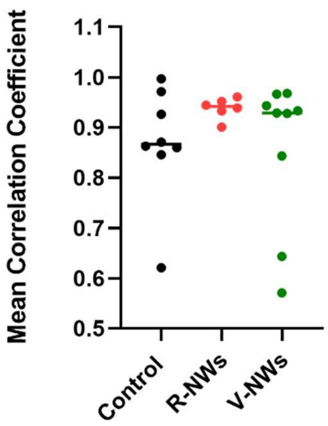

Figure S1. Synchronization analysis. Mean correlation coefficient of calcium transients in cultured neurons in a control (n=8 samples), R-NWs (n=5 samples), and V-NWs (n=6 samples) substrate.

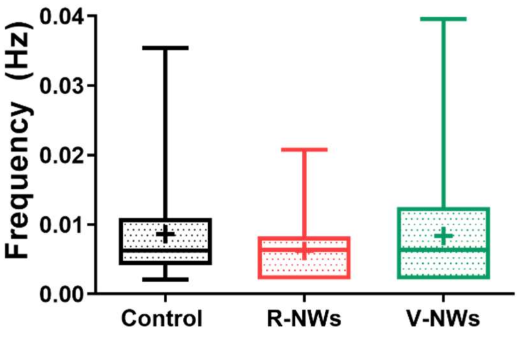

Figure S2. Box plot frequency for TTX in control (n=172 cells, 7 samples), R-NWs (118 cells, 4 samples) and V-NWs substrates (n = 73, 4 samples).
